# Metabolic Deregulation in Pulmonary Hypertension

**DOI:** 10.3390/cimb45060309

**Published:** 2023-06-03

**Authors:** Rajamma Mathew, Sanda Iacobas, Jing Huang, Dumitru Andrei Iacobas

**Affiliations:** 1 Department of Pediatrics, New York Medical College, Valhalla, NY 10595, USA; rmathew@nymc.edu; 2 Department of Physiology, New York Medical College, Valhalla, NY 10595, USA; 3 Department of Pathology, New York Medical College, Valhalla, NY 10595, USA; sandaiacobas@gmail.com; 4 Department of Pathology and Laboratory Medicine, Rutgers University Biomedical and Health Sciences, New Brunswick, NJ 08901, USA; jh1502@rutgers.edu; 5 Personalized Genomics Laboratory, Texas Undergraduate Medical Academy, Prairie View A&M University, Prairie View, TX 77446, USA

**Keywords:** Cav1, citrate cycle, fructose and mannose pathway, glycolysis/gluconeogenesis pathway, Ngf, Nfe2l2, PAH channelopathy, Pmm2, pyruvate metabolism, Slc2a1

## Abstract

The high morbidity and mortality rate of pulmonary arterial hypertension (PAH) is partially explained by metabolic deregulation. The present study complements our previous publication in “Genes” by identifying significant increases of the glucose transporter solute carrier family 2 (Slc2a1), beta nerve growth factor (Ngf), and nuclear factor erythroid-derived 2-like 2 (Nfe2l2) in three standard PAH rat models. PAH was induced by subjecting the animals to hypoxia (HO), or by injecting with monocrotaline in either normal (CM) or hypoxic (HM) atmospheric conditions. The Western blot and double immunofluorescent experiments were complemented with novel analyses of previously published transcriptomic datasets of the animal lungs from the perspective of the Genomic Fabric Paradigm. We found substantial remodeling of the citrate cycle, pyruvate metabolism, glycolysis/gluconeogenesis, and fructose and mannose pathways. According to the transcriptomic distance, glycolysis/gluconeogenesis was the most affected functional pathway in all three PAH models. PAH decoupled the coordinated expression of many metabolic genes, and replaced phosphomannomutase 2 (Pmm2) with phosphomannomutase 1 (Pmm1) in the center of the fructose and mannose metabolism. We also found significant regulation of key genes involved in PAH channelopathies. In conclusion, our data show that metabolic dysregulation is a major PAH pathogenic factor.

## 1. Introduction

Pulmonary arterial hypertension (PAH) is a rare disease with a high morbidity and mortality rate. Several systemic and genetic diseases, lung developmental defects, congenital heart defects, and drug toxicity are known to lead to PAH. Survival time in patients with PAH without treatment is reported to be about 2.8 years. Although modern treatment has slowed the progression of the disease, it has not halted it [1,2,3].

During the 6th World Symposium on Pulmonary Hypertension (WSPH), PAH was defined as the mean pulmonary artery pressure > 20 mmHg and pulmonary vascular resistance > 3 Wood units [4]. Irrespective of the underlying malady, endothelial cell (EC) dysfunction plays a vital role in the pathophysiology of PAH. EC dysfunction leads to impaired bioavailability of nitric oxide, activation of proliferative pathways, medial hypertrophy, neointima formation, and obstruction in the pulmonary arteries leading to increased pulmonary artery pressure, subsequent right ventricular hypertrophy, and heart failure resulting in premature death [5].

The disruption of endothelial caveolin-1 (Cav1), a cell membrane protein, has been shown to play a key role in the initiation and progression of pulmonary hypertension (PH) in experimental models, and also in human PAH. The loss of endothelial Cav1 is followed by an enhanced expression of Cav1 in smooth muscle cells, accompanied by a loss of caveolae. This Cav1 is tyrosine-phosphorylated, which is thought to participate in cell proliferation and migration. Significantly, a reduced number of caveolae has been observed when the smooth muscle cells shift from a contractile to a proliferative (synthetic) phenotype. These changes lead to further cell proliferation, medial hypertrophy, and neointima formation. Importantly, neointimal cells in experimental models and human PAH exhibit a lack of Cav1 [6,7,8,9].

The proliferative and obstructive vasculopathy of PAH is accompanied by a shift to aerobic glycolysis and mitochondrial fragmentation. Metabolic alterations, including glycolysis, increased glutamine utilization, and decreased fatty acid oxidation, observed in human and experimental models of PAH are reminiscent of cancer. In cancer, hypoxia-inducible factor-1α (HIF-1α) mediates effects such as metabolic shift, production of reactive oxygen species, inhibition of fatty acid β-oxidation, and alteration in the expression of tumor suppressor genes promote tumor progression [10]. In PAH, acquired mitochondrial abnormalities, such as epigenetic silencing of superoxide dismutase (*SOD2*), disrupt oxygen sensing, creating a pseudohypoxic environment characterized by normoxic activation of HIF-1α. The resulting metabolic shift to aerobic glycolysis (the Warburg phenomenon) reflects inhibition of pyruvate dehydrogenase by pyruvate dehydrogenase kinases [11]. In addition, *Cav1* knockdown in endothelial cells (in vitro studies) revealed a decrease in glycolytic intermediates and an increase in fatty acids indicative of metabolic switch [12]. An intact plasma membrane is required for compartmentation of glycolysis and gluconeogenesis. Disruption of caveolae results in the inhibition of glycolysis and stimulation of gluconeogenesis [13]. Furthermore, the expression of glucose transporter solute carrier family 2 member 1 (Slc2a1) has been shown to be increased in cancer; it is thought to regulate glycolysis and proliferation in cancer cells [14].

In hypoxia-induced PAH, endothelial Cav1 dysfunction was shown to be associated with the activation of proliferative pathways, loss of phosphatase and tensin homologue (*Pten*), and increased expression of *Slc2a1* [15]. In thyroid cancer cells, the loss of *PTEN* expression was found to be accompanied by increased expression of *SLC2A1* on the plasma membrane [16]. Caveolin-1 protein expression has been shown to determine the membrane-associated *PTEN* levels and activity. *PTEN* via the caveolin-1 binding sequence suppresses cell proliferation and, thus, maintains homeostasis [17]. Nuclear factor erythroid-derived 2-like 2 (Nfe2l2, also known as *Nrf2*) plays an important role in regulating the cellular redox status and metabolic reprogramming. Cav1 interacts with Nfe2l2 in the cytosol as well as in the nucleus. Caveolin-1 inhibits cellular antioxidant capacity through direct interaction and suppression of Nfe2l2, and Cav1 knockdown leads to dissociation between Nfe2l2 and its cytoplasmic inhibitor Keap1 (Kelch-like ECH-associated protein 1), thus increasing Nfe2l2 transcriptional activity [18,19]. Under normal conditions, Nfe2l2 protects cells against oxidative stress by activating genes encoding detoxifying and antioxidant proteins. However, Nfe2l2 accumulation has been shown to confer growth and survival in cancer [20,21]. In human PAH and experimental models of PAH, increased expression of nerve growth factor (*NGF*) has been reported. NGF promotes cell proliferation, migration, and increased activity of proinflammatory cytokines. Furthermore, Nfe2l2 was confirmed as the downstream target of *NGF* to promote viability, adhesion, and migration [22,23,24].

In a previous paper [25], we reported altered *Cav1* expression in the lungs of all three investigated PAH rat models. In order to test our hypothesis that metabolic dysregulation has an important role in PAH pathogenesis, in this new study performed on the same rat models, we examined alterations in the protein levels of Ngf, Nfe2l2, Slc2a1, and the restructuring of several metabolic pathways. In [25], we also presented the weight gain, right ventricle hypertrophy, right ventricle systolic pressure, and photos of the histology of pulmonary arteries in the same rat models of PAH.

Numerous publications have reported transcriptomic alterations caused by PAH in lungs from humans (e.g., [1,26]) and animal models of PAH ([27,28]), but they are limited to comparing only the average expression levels of genes in lungs of PAH and healthy subjects. Our study goes further by adopting the Genomic Fabric Paradigm (GFP) [29] that takes full advantage of the simultaneous profiling of tens of thousands of genes on several biological replicates. Thus, in addition to the average expression level (AVE), GFP attaches to every gene in each condition the independent relative expression variability (REV) among biological replicates and expression correlation (COR) with each other gene (19,999 CORs if 20,000 genes were quantified). By doing so, GFP approaches the cellular transcriptome as a mathematical object with hundreds of millions of dimensions, subjected to dynamic sets of transcription controls and expression correlations. GFP increases the amount of workable transcriptomic data obtained from a high-throughput (RNA sequencing or microarray) platform by four orders of magnitude without increasing the experimental costs. Thus, profiling the expressions of 20,000 genes yields 20,000 AVEs + 20,000 REVs + 199,990,000 (= 20,000 × (20,000 − 1)/2) CORs = 200,003,000 values, i.e., about 10,000× more workable information than considering only the AVEs.

REV shows how sensitive the transcription of that gene is to the slight (not significantly regulating) environmental differences among the biological replicates. As such, REV is in an inverse relationship with the strength of the transcription control exerted by the cellular homeostatic mechanisms to limit the expression random fluctuations. Therefore, REV analysis indicates the genes whose correct expression levels are critical for the cell’s life [30]. By comparing the REVs in PAH models with the CO group, one learns how each treatment triggering pulmonary arterial hypertension altered the cellular homeostatic mechanisms that control the transcript abundances.

Distinct from the traditional cluster analysis that groups the genes according to their coregulation when comparing two conditions, the COR analysis identifies the genes whose expression fluctuates in-phase or anti-phase across biological replicates of the SAME condition. COR analysis is used to refine the gene networking based on the “Principle of Transcriptomic Stoichiometry” (PTS) [31], a generalization of Dalton’s law from chemistry that imposes the correlated expression of genes whose encoded products are involved in a functional pathway. It also identifies the genes that are independently expressed, meaning that the encoded products do not interact in any functional pathway of the analyzed condition. By comparing the COR results in two conditions, one can determine and quantify the remodeling of the gene networks in functional pathways.

The genomic fabric of a functional pathway is, by definition, the transcriptome associated to the most intercoordinated and stably expressed gene network responsible for that functional pathway.

Several genes that control the mitochondrial membrane potential, and whose regulation is responsible for heart channelopathies [30], may also play mechanistic roles in triggering PAH. Therefore, in the present study, we examined the expression and contribution to the transcriptomic alteration in the PAH lung of the genes: *Abcc8*, *Abcc9*, *Kcna5*, *Kcnj8*, *Kcnk3*, *Trpc1*, *Trpc4*, *Trpc6*, and *Tymp.*

## 2. Materials and Methods

### 2.1. Animals

Sprague–Dawley rats (weight 150–175 g) were obtained from Charles River Laboratories (Wilmington, MA, USA). Rats were allowed to acclimatize in the animal facility for five days before the start of experimental protocols and had free access to chow and water. Protocol (“Mechanism of neointima formation in pulmonary hypertension”) was approved by the Institutional Animal Care and Use Committee of New York Medical College (approval Letter# 4-1-0113/2014, Banner No: 171020) and respected the guiding principles for the use and care of laboratory animals of the American Physiological Society and the National Institutes of Health.

Rats were divided into 4 groups: Gr.1 control (CO), Gr.2 monocrotaline (CM), Gr.3 hypoxia (HO), and Gr.4 monocrotaline + hypoxia (HM). Control rats (Gr.1) were maintained in room air, Gr.2 rats received monocrotaline (40 mg/kg, sc) and were kept in room air, Gr.3 rats were subjected to hypobaric hypoxia (atmospheric pressure 380 mmHg) starting on day 1, and Gr.4 rats received monocrotaline 40 mg/kg and were subjected to hypobaric hypoxia starting on day 1. The hypoxia chamber was opened twice per week for 15 min to weigh the rats, replenish food and water, and to provide clean bedding similar to the other rats in room air. At the end of the 4 weeks, the rats were sacrificed and their tissues were analyzed.

### 2.2. Hemodynamic Data

As previously described [6,7,8], rats were anesthetized with pentobarbital (60 mg/kg, ip), ventilated at 70–80 breaths/min through a tracheostomy, and cannulated with a PE240 tubing (tidal vol. 0.83 mL/100 g body weight). A thoracotomy was performed and the right ventricular systolic pressure (RVSP) was measured with a small needle attached to a PE50 tubing, using a Grass polygraph (model 7E). After perfusing the lungs with normal saline, heart and lungs were removed. The ratio of the right ventricle (RV) and the left ventricle including septum (LV) was used to assess right ventricular hypertrophy (RVH). The right lung was frozen and stored at −80 °C. The heart and the left lung were kept in 10% buffered formaldehyde.

### 2.3. Estimation of Protein Expression

Western blot analysis was carried out as previously described [6,7,8]. Briefly, the lung tissue was homogenized in a buffer containing 0.1 M PBS (pH 7.4), 0.5% sodium deoxycholate, 1% Igepal, 0.1% SDS, 10 mL/mL phenylmethylsulfonic fluoride (PMSF; 10 mg dissolved in 1 mL of isopropanol), 25 mg/mL aprotinin, and 25 mg/mL leupeptin. PMSF (10 mL/mL) and phosphatase Inhibitor Cocktail 1 (10 mL/mL; Sigma, St. Louis, MO, USA) were added to homogenates placed on ice for 30 min, and then centrifuged at 14,000 rpm for 20 min at 40 °C. A total of 50 or 100 mg of protein from lung supernatants was loaded and separated on a 10% sodium SDS-polyacrylamide gel (Mini Protean-II, Bio-Rad, Hercules, CA, USA) and transferred to nitrocellulose membrane (Hybond ECL, Amersham Life Science, Boston, MA, USA) using Semi-Dry Transfer Cell (Bio-Rad). The membranes were blocked with 5% nonfat milk powder in Tris-Buffered Saline with Tween buffer (TBST; 10 mM Tris-HCl, pH 7.4, 150 mM NaCl, 0.05% Tween 20) overnight at 40 C. Membranes were then incubated with antibodies of interest: Slc2a1 (1:400, Abcam, Abcam), Nfe2l2 (1:400 Abcam, Cambridge, UK), or NGF (1:800, Santa Cruz, Santa Cruz, CA, USA) for 1 h at room temperature, or overnight at 40 C, according to the manufacturers’ recommendations. The blots were reprobed with β-actin (1:10,000) to assess the protein loading. The protein bands were visualized by chemiluminescence (ECL Western Blotting Analysis System, Amersham International, Amersham, UK). The relative expressions of the proteins were quantified using densitometric scanning, and were expressed as the ratio of protein of interest and β-actin.

### 2.4. Double Immunofluorescence

Double immunofluorescence was carried out as described previously [25]. Briefly, lung sections were mounted on superfrost plus microscope slides (VWR Scientific, Radnor, PA, USA) and deparaffinized in xylene (5 min ×2), rehydrated through a range of aqueous ethyl alcohol solutions in H_2_O, and immersed in PBS for 5 min. Antigen retrieval was performed by incubating the sections in 10 mM citrate buffer, pH 6, in a microwave oven for 5 min. The slides were then incubated in a blocking solution (5% normal donkey serum, 0.5% Triton in PBS) for 1 h at room temperature, followed by an overnight incubation at 4 °C with the primary antibody Slc2a1 (1:400) in blocking solution. The next day, the slides were washed with PBS for 10 min ×3 and incubated in Alexa 488 (donkey anti-rabbit, 1:300, green color, Molecular Probes) at room temperature in a dark place for 1 h, followed by washing in PBS for 10 min ×3. For double staining, the sections were blocked again, as described earlier, and incubated in the smooth muscle α-actin antibody (1:15) overnight; the procedure was repeated using the appropriate secondary antibody Alexa 594 (donkey anti-rabbit 1:200, red color, Molecular Probes, Eugene, OR, USA). The sections were examined with a laser scanning confocal fluorescence microscope (MRC 1000, Bio-Rad). The negative controls were run in the absence of primary antibodies.

### 2.5. Statistical Analysis

The data are expressed as means ± SEM. For statistical analysis, we used one-way analysis of variance followed by Scheffe’s multiple comparison tests. Differences were considered statistically significant at *p* < 0.05.

### 2.6. Gene Expression Data

This study used the gene expression results deposited and publicly available at https://www.ncbi.nlm.nih.gov/geo/query/acc.cgi?acc=GSE72707 (accessed on 1 April 2023). The data were obtained by profiling the lungs of the three rat models using Agilent 60 mer 4 × 44 k whole genome rat V2 arrays (#G2519F), as previously described [25]. Filtered raw data were further processed and the reciprocally independent characteristics AVE (average expression level), REV (relative expression variability), and COR (expression correlation with each other gene) were attached to every single gene in each condition.

Because numerous genes are probed redundantly in the Agilent microarray by more than one spot (nonuniform number of spots), the three independent types of characteristics were computed as:

(1) $$\begin{array}{l} AVE_{i}^{\left(condition\right)}=\frac{1}{R_{i}}\sum_{k=1}^{R_{i}}\mu_{i,k}^{\left(condition\right)}=\frac{1}{R_{i}}\sum_{k=1}^{R_{i}}\left(\frac{1}{4}\sum_{j=1}^{4}a_{i,k,j}^{\left(condition\right)}\right)\;,\;where:\\ condition=“CO”,“CM”,“HO”,“HM”\\ R_{i}=\;\mathrm{number}\;\mathrm{of}\;\mathrm{spots}\;\mathrm{probing}\;\mathrm{redundantly}\;\mathrm{gene}\;“i”,\\ a_{i,k,j}^{\left(condition\right)}=\mathrm{normalized}\;\mathrm{expression}\;\mathrm{level}\;\mathrm{of}\;\mathrm{gene}\;“i”\;\mathrm{probed}\;\mathrm{by}\;\mathrm{spot}\;“k”\;\\ \;\;\;\;\;\;\mathrm{on}\;\mathrm{biological}\;\mathrm{replica}\;“j”\;\mathrm{in}\;“\mathrm{condition}” \end{array}$$

The expression levels in each condition were normalized to the median expression level of all genes in that condition.

(2) $$\begin{array}{l} REV_{i}^{\left(condition\right)}=\underset{\mathrm{chi}-\mathrm{square}\;\mathrm{mid}-\mathrm{interval}\;\mathrm{estimate}\;\mathrm{of}\;\mathrm{the}\;\mathrm{coefficient}\;\mathrm{of}\;\mathrm{variation}}{\underbrace{\frac{1}{2}\left(\sqrt{\frac{r_{i}}{\chi^{2}\left(r_{i};0.975\right)}}+\sqrt{\frac{r_{i}}{\chi^{2}\left(r_{i};0.025\right)}}\right)}}\sqrt{\underset{\mathrm{pooled}\;\mathrm{CV}\;\mathrm{for}\;\mathrm{all}\;\mathrm{spots}\;\mathrm{probing}\;\mathrm{gene}\;i}{\underbrace{\frac{1}{R_{i}}\sum_{k=1}^{R_{i}}{\left(\frac{s_{ik}^{\left(condition\right)}}{\mu_{ik}^{\left(condition\right)}}\right)}^{2}}}}\times 100\%\\ \chi^{2}\left(r_{i};\alpha \right)=\mathrm{chi}-\mathrm{square}\;\mathrm{for}\;r_{i}(=4R_{i}-1=\;\mathrm{number}\;\mathrm{of}\;\mathrm{degrees}\;\mathrm{of}\;\mathrm{freedom})\;\mathrm{and}\;\mathrm{probability}\;\alpha \\ \mu_{ik}=\mathrm{average}\;\mathrm{expression}\;\mathrm{of}\;\mathrm{gene}\;i\;\mathrm{probed}\;\mathrm{by}\;\mathrm{spot}\;k\;(=1,\;\ldots ,\;R_{i})\;\mathrm{in}\;\mathrm{the}\;4\;\mathrm{biological}\;\mathrm{replicas}\\ s_{ik}=\mathrm{standard}\;\mathrm{deviation}\;\mathrm{of}\;\mathrm{the}\;\mathrm{expression}\;\mathrm{level}\;\mathrm{of}\;\mathrm{gene}\;i\;\mathrm{probed}\;\mathrm{by}\;\mathrm{spot}\;k \end{array}$$

Lower REV values indicate genes whose random fluctuations of the expression level are strongly limited by the cellular homeostatic mechanisms; such genes are most likely critical for cell survival, phenotypic expression, and proliferation. By contrast, higher REVs are associated with less-controlled genes that may function as vectors of cell adaptation to the environmental fluctuations [32].

COR analysis is based on the Pearson product–moment correlation coefficient between the (log_2_) expressions of each gene *i* across biological replicates with each other gene *g* in the same group of replicates [30].

(3) $$COR_{ig}^{\left(condition\right)}=\frac{\sum_{k_{i}=1}^{R_{i}}\sum_{k_{g}=1}^{Rg}\left(\sum_{j=1}^{4}\left(a_{i,k,j}^{\left(condition\right)}-AVE_{i}^{\left(condition\right)}\right)\left(a_{g,k,j}^{\left(condition\right)}-AVE_{g}^{\left(condition\right)}\right)\right)}{\sqrt{\sum_{k_{i}=1}^{R_{i}}\left(\sum_{j=1}^{4}{\left(a_{i,k,j}^{\left(condition\right)}-AVE_{i}^{\left(condition\right)}\right)}^{2}\right)\sum_{k_{g}=1}^{R_{g}}\left(\sum_{j=1}^{4}{\left(a_{g,k,j}^{\left(condition\right)}-AVE_{g}^{\left(condition\right)}\right)}^{2}\right)}}$$

COR analysis identifies the (*p* < 0.05) significantly synergistically (in-phase fluctuations), antagonistically (opposite fluctuations), and independently expressed gene pairs in each condition. One can further determine the coordination score COORD as:

(4) $$COORD_{\Gamma }^{\left(condition\right)}(p)=SYN_{\Gamma }^{\left(condition\right)}(p)+ANT_{\Gamma }^{\left(condition\right)}(p)-IND_{\Gamma }^{\left(condition\right)}(p)$$

where *SYN*, *ANT*, and *IND* are the percentages of the gene pairs that are *p*-significantly synergistically, antagonistically, or independently expressed within the investigated functional pathway Γ in the given *condition.*

COR refines the gene wiring in functional pathways built by specialized software, such as Ingenuity [33], DAVID [34], KEGG [35], etc., that ignore the race/strain, sex, age, and other factors known to influence the incidence of the disease, and the response to treatment. By combining REV and COR, GFP singles out for each patient, in each condition, the most stably expressed and interconnected gene network responsible for that particular biological process.

### 2.7. Transcriptomic Analyses

#### 2.7.1. Significant Regulation

Traditional analysis considers a gene as significantly regulated in the disease, with respect to the healthy counterpart, when the absolute expression ratio (|x|) exceeds an arbitrarily introduced cutoff (e.g., 1.5x or 2.0x) and/or the *p*-value of the heteroscedastic *t*-test of the means’ equality is less than 0.05 or 0.01 (another arbitrarily introduced cutoff criterion). While we are kipping the *p*-value condition, for the expression ratio we determine for each gene, in every pair of conditions to be compared, the cutoff “CUT” that takes into account both the biological variability of that gene in both conditions and the technical noise of the probing spot(s) in the two groups of 4 microarrays. Thus, for stably expressed genes (low variability among biological replicates) and accurate probing (low technical noise), CUT is below 1.5, while for others it may exceed 1.5.

(5) $$\begin{array}{l} \forall PH=HO,CM,HM\\ p_{i}^{\left(CO\to PH\right)}<0.05\\ \left|x_{i}^{\left(CO\to PH\right)}\right|>CUT_{i}^{\left(CO\to PH\right)}=1+\frac{1}{100}\sqrt{2\left({\left(REV_{i}^{\left(CO\right)}\right)}^{2}+{\left(REV_{i}^{\left(PH\right)}\right)}^{2}\right)}\;,\;where:\\ \;\;x_{i}^{\left(CO\to PH\right)}=\left\{\begin{array}{lll} \sum_{k=1}^{R_{i}}\mu_{ik}^{\left(PH\right)}/\sum_{k=1}^{R_{i}}\mu_{ik}^{\left(CO\right)} & if & \sum_{k=1}^{R_{i}}\mu_{ik}^{\left(PH\right)}\geq \sum_{k=1}^{R_{i}}\mu_{ik}^{\left(CO\right)}\\ -\sum_{k=1}^{R_{i}}\mu_{ik}^{\left(CO\right)}/\sum_{k=1}^{R_{i}}\mu_{ik}^{\left(PH\right)} & if & \sum_{k=1}^{R_{i}}\mu_{ik}^{\left(PH\right)}<\sum_{k=1}^{R_{i}}\mu_{ik}^{\left(CO\right)} \end{array}\right. \end{array}$$

#### 2.7.2. Measures of Individual Genes’ Contributions to the Transcriptomic Alteration

In addition to the traditional percentage of significantly regulated (PSR) genes and expression ratio (x), we use the “Weighted Individual (gene) Regulation (WIR) and the 3D vector “Individual (gene) Transcriptomic Trajectory” (ITT), whose module is the “Transcriptomic Distance” (TD). PSR is restricted to the significantly regulated genes (according to arbitrarily introduced criteria) and implicitly considers all genes as uniform +1 or −1 contributors to the overall transcriptomic alteration. In contrast, WIR and TD take into account all genes. While WIR weights the contribution of the AVE change by the normal expression level, the net absolute fold-change, and the confidence of the expression regulation, TD goes further by also quantifying the changes in expression variability and expression coordination with all other genes.

(6) $$WIR_{i}^{\left(CO\to PH\right)}=\underset{\mathrm{departure}\;\mathrm{from}\;\mathrm{the}\;\mathrm{normal}\;\mathrm{level}}{\underbrace{\mu_{i}^{\left(CO\right)}\left(\left|x_{i}^{\left(CO\to PH\right)}\right|-1\right)}}\frac{x_{i}^{\left(CO\to PH\right)}}{\left|x_{i}^{\left(CO\to PH\right)}\right|}\underset{\mathrm{confidence}\;\mathrm{of}\;\mathrm{regulation}}{\underbrace{\left(1-p_{i}^{\left(CO\to PH\right)}\right)}}\;$$

(7) $$\begin{array}{l} 3D\;\mathrm{vector}\;\mathrm{with}\;\mathrm{orthogonal}\;\mathrm{components}\\ \vec{ITT_{i}^{\left(CO\to PH\right)}}:\left(\frac{AVE_{i}^{\left(PH\right)}-AVE_{i}^{\left(CO\right)}}{{\langle AVE_{i}^{\left(CO\right)}\rangle }_{all\;i}}\right),\left(\frac{REV_{i}^{\left(PH\right)}-REV_{i}^{\left(CO\right)}}{{\langle REV_{i}^{\left(CO\right)}\rangle }_{all\;i}}\right),\sqrt{\frac{{\langle {\left(COR_{i,j}^{\left(PH\right)}-COR_{i,j}^{\left(CO\right)}\right)}^{2}\rangle }_{all\;j}}{{\langle {\left(COR_{i,j}^{\left(CO\right)}\right)}^{2}\rangle }_{all\;j}}}\\ {\langle Y_{i}^{\left(CO\right)}\rangle }_{\mathrm{gene}\;\mathrm{subset}}=\mathrm{average}\;\mathrm{characteristic}\;“Y”\;\mathrm{over}\;a\;\mathrm{gene}\;\mathrm{subset}\;\mathrm{in}\;\mathrm{condition}\;“\mathrm{CO}”\;\\ “\mathrm{transcriptomic}\;\mathrm{distance}”\;\mathrm{to}\;\mathrm{reference}\;\mathrm{condition}\;\mathrm{CO}\;(\mathrm{origin}):\\ \underset{\left|\vec{ITT_{i}^{\left(CO\to PH\right)}}\right|}{\underbrace{TD_{i}^{\left(CO\to PH\right)}}}\equiv \sqrt{{\left(\frac{AVE_{i}^{\left(PH\right)}-AVE_{i}^{\left(CO\right)}}{{\langle AVE_{i}^{\left(CO\right)}\rangle }_{all\;i}}\right)}^{2}+{\left(\frac{REV_{i}^{\left(PH\right)}-REV_{i}^{\left(CO\right)}}{{\langle REV_{i}^{\left(CO\right)}\rangle }_{all\;i}}\right)}^{2}+\frac{{\langle {\left(COR_{i,j}^{\left(PH\right)}-COR_{i,j}^{\left(CO\right)}\right)}^{2}\rangle }_{all\;j}}{{\langle {\left(COR_{i,j}^{\left(CO\right)}\right)}^{2}\rangle }_{all\;j}}} \end{array}$$

## 3. Results

### 3.1. Significant Increase of the RVSP and RV/LV Weight Ratio in All Three PAH Models

RVSP and RV/LV weight ratio in the control group (CO) were: 21 ± 0.8 mmHg and 0.23 ± 0.004, respectively. RVSP was significantly increased in all PAH groups: 53 ± 3.48* mmHg in HO, 66 ± 8* mmHg in CM, and 90 ± 0.104** mmHg in HM. RV/LV ratio was also significantly increased to: 0.56 ± 0.004* in HO, 0.65 ± 0.004* in CM, and 0.65 ± 0.004* in HM (* = *p* < 0.05 significant, ** = *p* < 0.01 significant).

### 3.2. Pulmonary Arterial Hypertension Increased the Expression of the Nerve Growth Factor (Ngf), Nuclear Factor Erythroid-Derived 2-Like 2 (Nfe2l2), and Glucose Transporter Solute Carrier Family 2 (Slc2a1)

**Figure 1:**
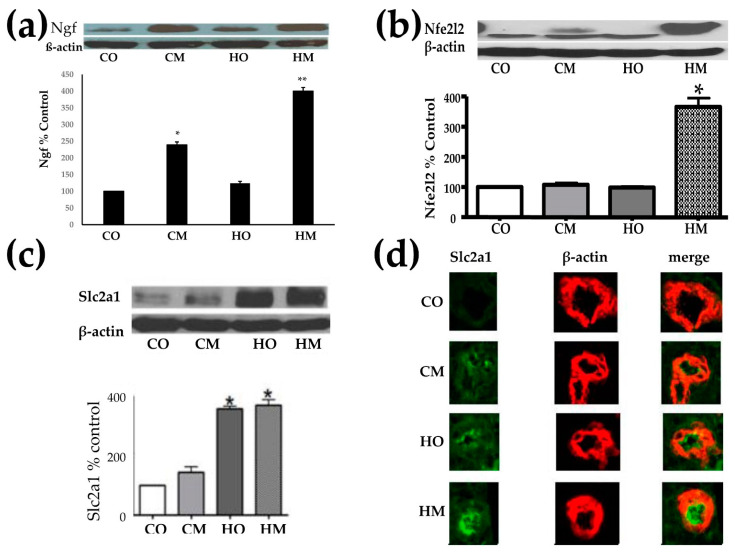
Western blot analysis of Ngf (**a**), Nfe2l2 (**b**), and Slc2a1 (**c**) in all groups of PAH rats with respect to controls. (**d**) Double immunofluorescence showing increased expression of Slc2a1 abundances in the lungs of the three rat PAH models with respect to the control group, although the increase in the CM group was not (*p* < 0.05) statistically significant. * *p* < 0.05, ** *p* < 0.01.

Figure 1 presents the Western blot analysis of the nerve growth factor (Ngf), nuclear factor erythroid-derived 2-like 2 (Nfe2l2), and glucose transporter solute carrier family 2 (Slc2a1) in all groups of PAH rats with respect to controls. Of note is the statistically (*p* < 0.05) significant increase of Ngf in CM and (*p* < 0.01) in HM. Although the HO group Ngf exhibited an average of 23.5% increase, it was not statistically significant. The data indicate that the administration of monocrotaline is the main driver of the Ngf increase, with hypoxia enhancing its effect.

Interestingly, only the combination of hypoxia exposure and monocrotaline administration produced a statistically significant increase of Nfe2l2.

The increase of Slc2a1 was statistically (*p* < 0.05) significant in both groups of hypoxia-exposed animals, but not in the CM group (rats injected with monocrotaline in normal atmospheric conditions).

### 3.3. Independent Transcriptomic Characteristics

**Figure 2:**
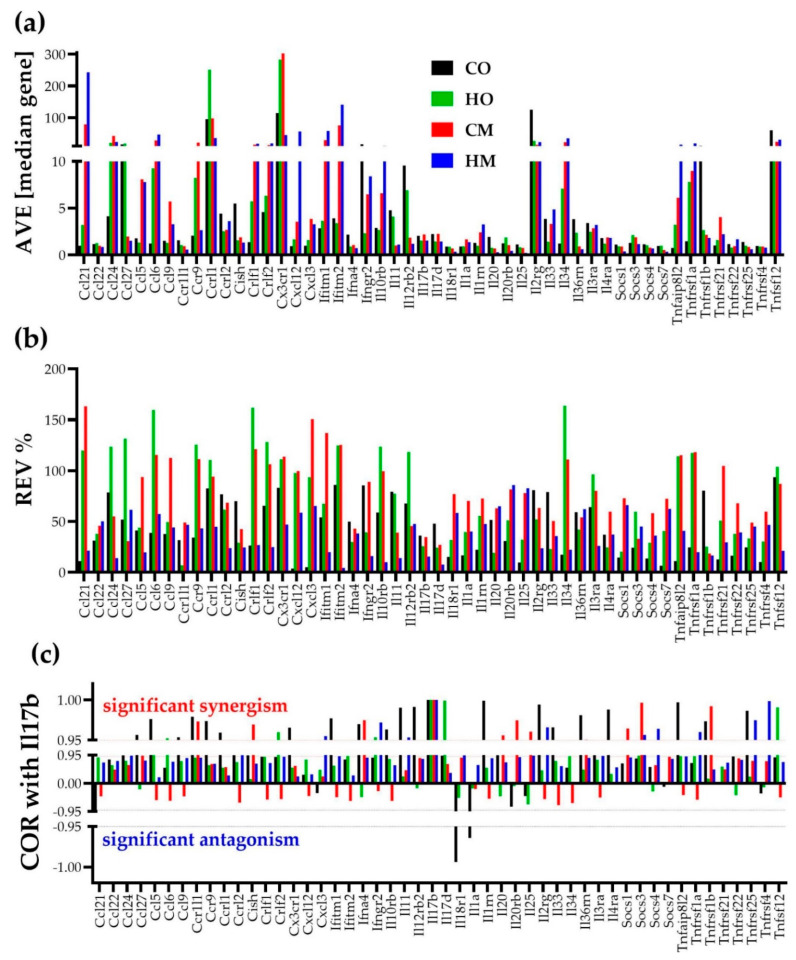
The independence of the (**a**) average expression level (AVE), (**b**) relative expression variability (REV), and (**c**) expression correlation (COR, here with Il17b) of 50 genes involved in the immune inflammatory response in the lungs of the control (CO) and PAH rat models (HO, CM, HM). The value 1 in panel (**c**) for Il17b in all conditions is a direct validation of the Pearson correlation coefficient. The correlation was considered as statistically significant if |COR| ≥ 0.95.

Figure 2 illustrates the independence of the three types of transcriptomic characteristics for 50 genes involved in the KEGG-determined immune response [36]. Correlation with interleukin 17B (Il17b) was selected for this illustration, owing to contrasting reports of both pro- and anti-inflammatory function of Il17b in rodents [37]. The impact of PAH on the immune response is important by itself; however, any other subset of genes would verify the independence of the three types of characteristics in any condition, as we proved in several other genomic studies on human cancers (e.g., [38]) and animal models of neurological diseases (e.g., [39]).

While the independence of the three characteristics is evident by visual inspection, also of note are the substantial differences both among the genes and among the conditions. Thus, within this section, AVE goes from 0.22 median gene expression for interleukin 25 (Il25) in HM (1.12 in CO, 0.82 in HO, and 0.75 in CM) to 302.56 median gene for chemokine (C-X3-C motif) receptor 1 (Cx3cr1) in CM (114.29 in CO, 283.17 in HO, and 45.20 in HM). REV goes from 3.71% for chemokine (C-X-C motif) ligand 12 (Cxcl12) in CO (97.67% in HO, 99.80% in CM, 58.78% in HM) to 159.57% for chemokine (C-C motif) ligand 6 (Ccl6) in HO (38.93% in CO, 115.51% in CM, 57.39% in HM).

The substantial synergistic expression correlation of this subset of genes with *Il17b* in CO (55.10%) was significantly reduced in the PAH models (22.45% in both HO and CM, and 32.65% in HM). Using the “CORRELATION” software(v.1) [40], we found that the (*p* < 0.05) significant coordination score within this 50-gene subset decreased from 31.51% in CO to 7.02% in HO, 22.04% in CM, and 24.37% in HM.

**Figure 3:**
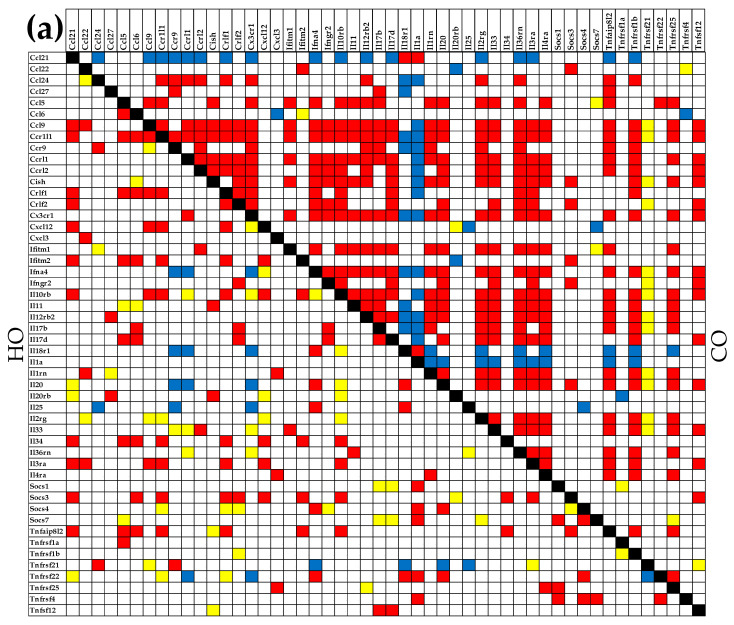

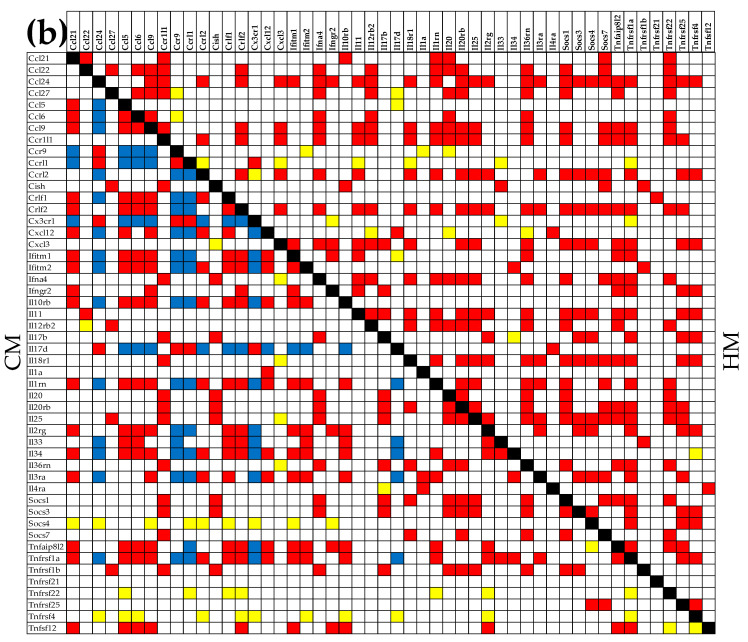
Significant synergistic, antagonistic, and independent pairing of 50 immune inflammatory response genes in the lungs of all animal groups. (**a**) CO and HO groups. (**b**) CM and HM grous. A red/blue/yellow square indicates that the genes labeling the intersecting row and column are synergistically/antagonistically/independently expressed in that designated condition, respectively. A blank square indicates no statistical significance of the expression correlation of the two genes.

Figure 3 shows the significant synergistic (28.00% in CO, 9.63% in HO, 18.45% in CM, 24.24% in HM), antagonistic (5.22% in CO, 1.55% in HO, 6.61% in CM, 0.00% in HM), and independent (1.71% in CO, 4.16% in HO, 3.02% in CM, 1.88% in HM) expression correlations in all four groups of rats for this gene subset. Of note are the differences on the significant correlations among the four groups of animals and the absence of antagonistic expressions in HM. For instance, the significant synergistic expression of Ccl21 and Ccl9 in CO was turned into a significant antagonistic expression in HO, while preserving the significant synergism in CM, and losing the statistical significance (albeit still synergistic) in HM.

### 3.4. Measures of Transcriptomic Alterations

**Figure 4:**
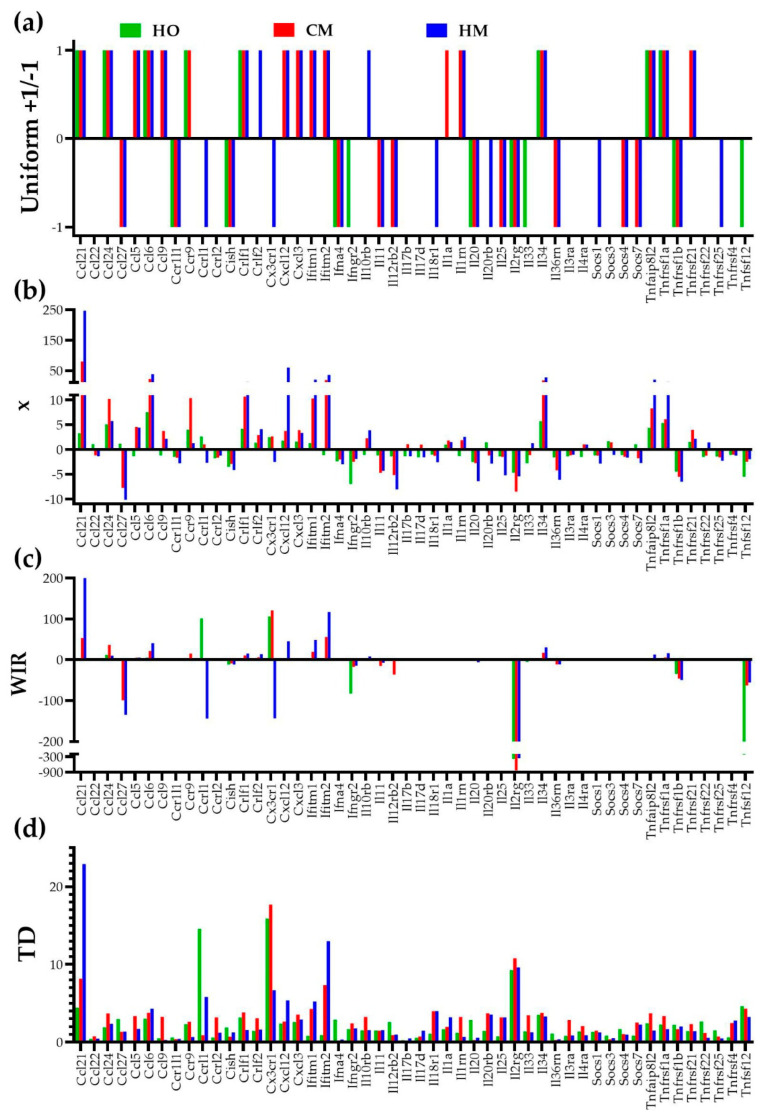
Four ways to quantify the contributions of the individual genes to the transcriptomic alterations in lungs of the PAH models illustrated by the regulation of 50 immune response genes. (**a**) Uniform—each significantly regulated gene is a +1 or −1 contributor, all other genes have no contribution. (**b**) Expression ratio x (negative for downregulation). (**c**) Weighted individual (gene) regulation (WIR). (**d**) Transcriptomic distance (TD) to that gene’s normal transcriptome (CO values for AVE, REV, and COR). Note how WIR and TD discriminate the genes according to their contributions to the overall alteration of the lung transcriptome.

Figure 4 presents the four quantification methods applied to 50 genes involved in the immune response. Most reports just count how many genes were significantly (according to arbitrarily introduced criteria) up- or downregulated, eventually listing the genes with the largest positive and negative fold-change. However, we believe that the analysis not only should include all genes, but should also go further by showing how much the change of the expression level counts for the expression profile of the entire transcriptome, as by WIR scoring. Given that PAH alters also the homeostatic mechanisms that limit the random expression fluctuations and remodel the gene networks, in addition to WIR, we also introduced the comprehensive quantifier “transcriptomic distance” to the normal state. Figure 4 illustrates our four ways to quantify the contributions of the individual genes to the transcriptomic alterations in the PAH models: uniform, expression ratio “x”, weighted individual (gene) regulation “WIR”, and transcriptomic distance “TD” to the normal/control state (CO).

### 3.5. Regulation of Glycolysis/Gluconeogenesis

**Figure 5:**
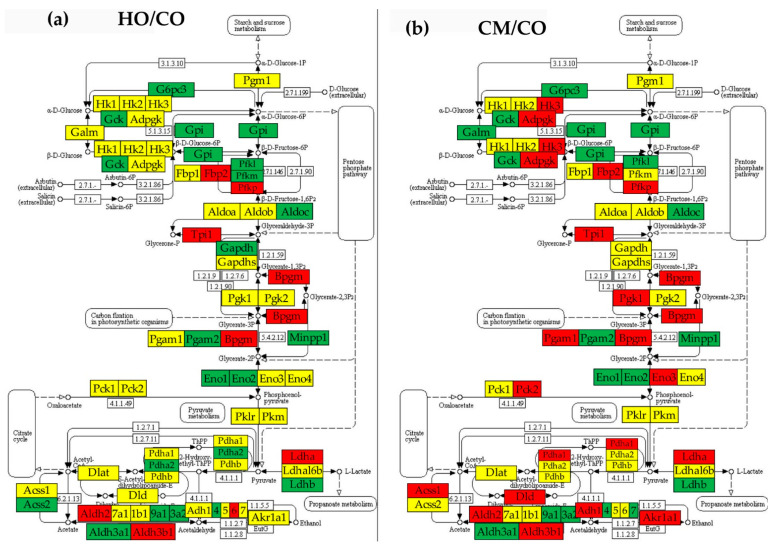

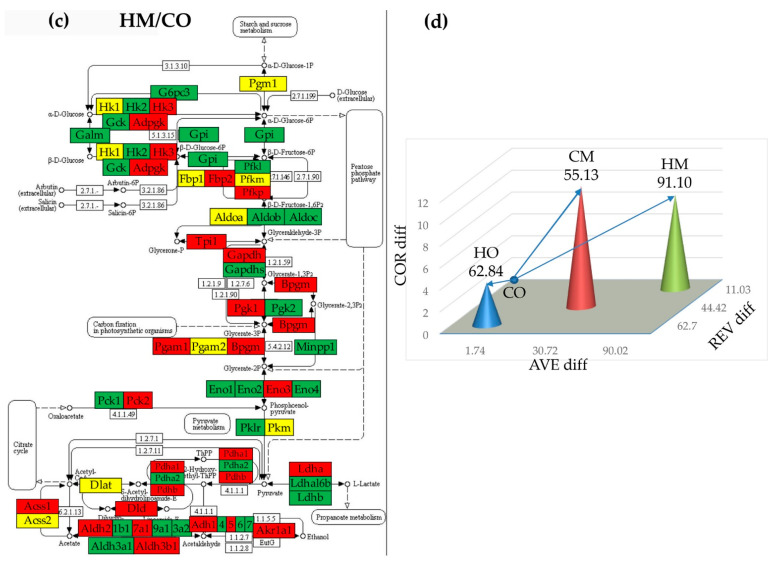
Regulation of the glycolysis/gluconeogenesis pathway in the PAH rat models with respect to control (CO). (**a**) Regulated genes in the HO model. (**b**) Regulated genes in the CM model. (**c**) Regulated genes in the HM model. (**d**) Transcriptomic distances separating the transcriptome associated with the glycolysis/gluconeogenesis pathway in the HO, CM, and HM PAH models from those in the control (CO) rats. Red/green/yellow background of the gene symbol indicates up-/down-/no regulation with respect to CO, respectively. Blank background blocks indicate no genetic information for that functional unit. Regulated genes: *Acss1/2* (acyl-CoA synthetase short-chain family member 1/2), *Adh1/4/5/6/7* (alcohol dehydrogenase 1/4/5/6/7), *Adpgk* (ADP-dependent glucokinase), *Aldh1b1* (aldehyde dehydrogenase 1 family, member B1), *Aldh2* (aldehyde dehydrogenase 2 family), *Aldh3a1/2* (aldehyde dehydrogenase 3 family, member A1/2), *Aldh3b1* (aldehyde dehydrogenase 3 family, member B1), *Aldh9a1* (aldehyde dehydrogenase 9 family, member A1), *Aldob/c* (aldolase B/C, fructose-bisphosphate), *Bpgm* (2,3-bisphosphoglycerate mutase), *Dld* (Dihydrolipoamide dehydrogenase), *Eno1/2/3/4* (enolase 1/2/3/4), *Fbp2* (fructose-1,6-bisphosphatase 2), *G6pc3* (glucose 6 phosphatase, catalytic, 3), *Galm* (galactose mutarotase (aldose 1-epimerase)), *Gapdh* (glyceraldehyde-3-phosphate dehydrogenase), *Gapdhs* (glyceraldehyde-3-phosphate dehydrogenase, spermatogenic), *Gck* (glucokinase), *Gpi* (glucose-6-phosphate isomerase), *Hk2/3* (hexokinase 2/3), *Ldha/b/c* (lactate dehydrogenase A/B/C), *Ldha16b* (lactate dehydrogenase A-like 6B), *Loc303448* (Ssimilar to glyceraldehyde-3-phosphate dehydrogenase), *Minpp1* (similar to glyceraldehyde-3-phosphate dehydrogenase), *Pck1/2* (phosphoenolpyruvate carboxykinase ½), *Pdha1/2* (pyruvate dehydrogenase (lipoamide) alpha ½), *Pdhb* (pyruvate dehydrogenase (lipoamide) beta), *Pfkl/m/p* (phosphofructokinase, liver/muscle/platelet), *Pgam1/2* (phosphoglycerate mutase ½), *Pgk1/2* (phosphoglycerate kinase ½), *Pklr* (pyruvate kinase, liver and RBC), *Tpi1* (triosephosphate isomerase 1). The tips of the colored cones in panel (**d**) are the coordinates of the pathway in the 3D pre-Hilbert space of states, while numbers on top of the cones are the average TDs of the composing genes in the indicated model.

Figure 5 shows the KEGG-determined glycolysis/gluconeogenesis metabolic pathway [41], indicating what genes were significantly regulated in the three PAH models (HO, CM, HM) and the transcriptomic distance with respect to CO.

### 3.6. Regulation of the Citrate (TCA) Cycle

**Figure 6:**
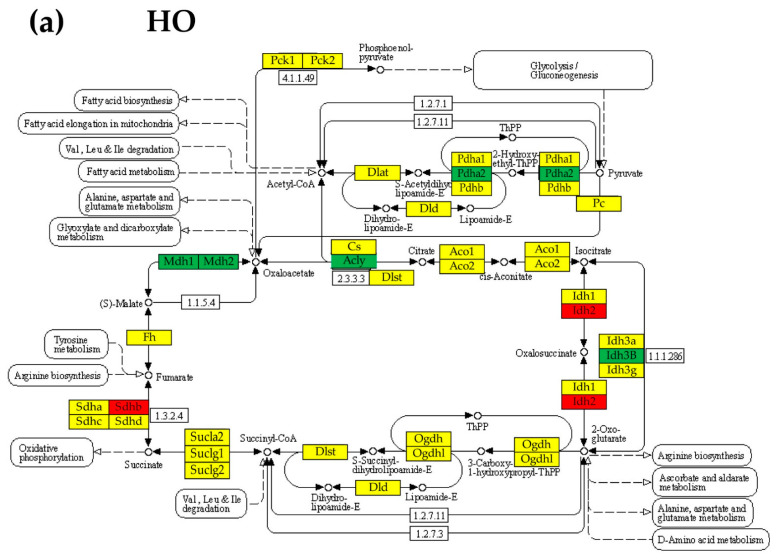

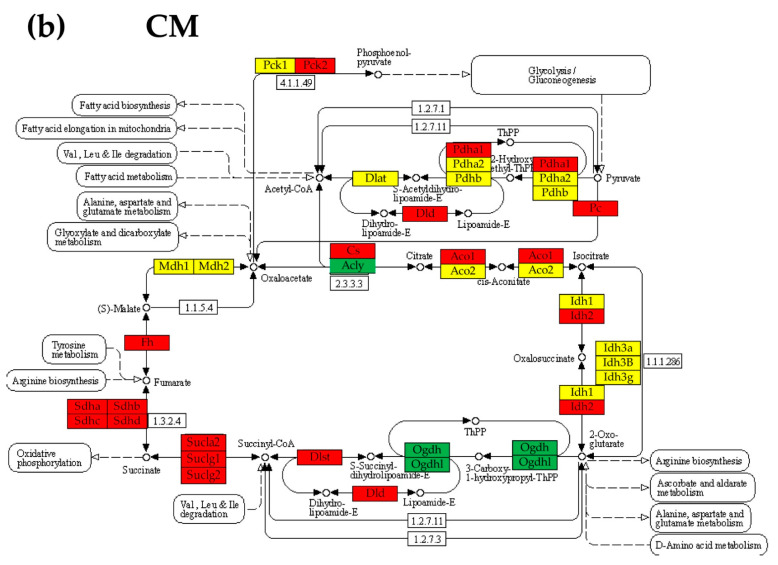

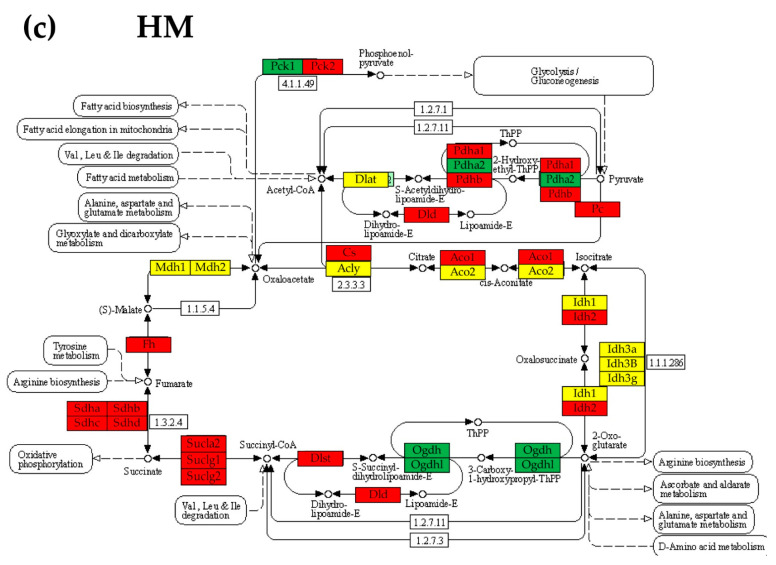
(*p* < 0.05) Significant regulation of the genes involved in the KEGG-determined citrate cycle in (**a**) HO group, (**b**) CM group, and (**c**) HM group with respect to CO group. Red/green/yellow background of the gene symbol indicates up-/down-/no regulation. White background in a block indicates that the genes from that group were not yet identified for rat. Note the differences among the three PAH models. Regulated genes: *Acly* (ATP citrate lyase), *Aco1* (aconitase 1, soluble), *Cs* (citrate synthase), *Dld* (dihydrolipoamide dehydrogenase), *Dlst* (dihydrolipoamide S-succinyltransferase (E2 component of 2-oxo-glutarate complex)), *Fh* (fumarate hydratase), *Idh2* (isocitrate dehydrogenase 2 (NADP+), mitochondrial), *Idh3b* (isocitrate dehydrogenase 3 (NAD+) beta), *Mdh1* (malate dehydrogenase 1, NAD (soluble)), *Mdh2* (malate dehydrogenase 2, NAD (mitochondrial)), *Ogdh* (oxoglutarate (alpha-ketoglutarate) dehydrogenase (lipoamide)), *Ogdhl* (oxoglutarate dehydrogenase-like), *PC* (pyruvate carboxylase), *Pck1* (phosphoenolpyruvate carboxykinase 1 (soluble)), *Pck2* (phosphoenolpyruvate carboxykinase 2 (mitochondrial)), *Pdha1* (pyruvate dehydrogenase (lipoamide) alpha 1), *Pdha2* (pyruvate dehydrogenase (lipoamide) alpha 2), *Pdhb* (pyruvate dehydrogenase (lipoamide) beta), *Sdha* (succinate dehydrogenase complex, subunit A, flavoprotein (Fp)), *Sdhb* (succinate dehydrogenase complex, subunit B, iron sulfur (Ip)), *Sdhc* (succinate dehydrogenase complex, subunit C, integral membrane protein), *Sdhd* (succinate dehydrogenase complex, subunit D, integral membrane protein), *Sucla2* (succinate-CoA ligase, ADP-forming, beta subunit), *Suclg1* (succinate-CoA ligase, alpha subunit), *Suclg2* (succinate-CoA ligase, GDP-forming, beta subunit).

Figure 6 presents the significant regulation of the genes involved in the KEGG-determined citrate cycle [42], an important aerobic pathway for the final steps of the oxidation of carbohydrates and fatty acids in the lungs of the three PAH models with respect to control. Out of the 30 genes quantified for this cycle, 7 (5 down, 2 up) were regulated in HO, 19 (3 down, 16 up) in CM, and 21 (4 down, 17 up) in HM, indicating that the monocrotaline treatment was the most altering factor.

### 3.7. Remodeling of the Metabolic Genes Network

**Figure 7:**
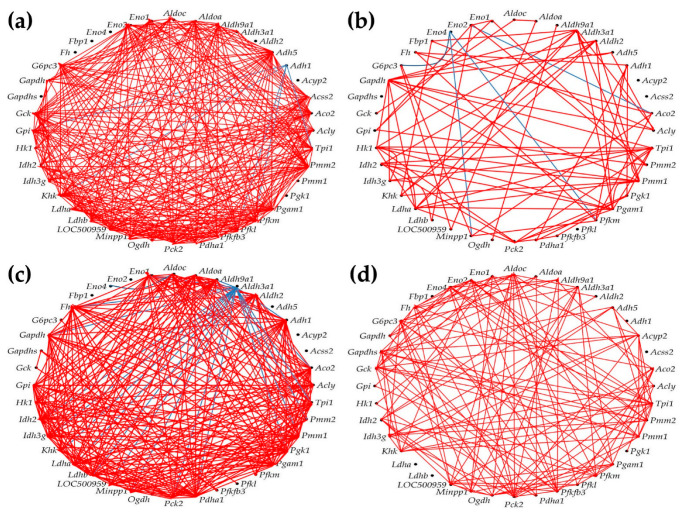
(*p* < 0.05) Significant synergistic (red lines) and antagonistic (blue lines) expression coordination of 40 selected metabolic genes in the four rat models. (**a**) CO. (**b**) HO. (**c**) CM. (**d**) HM. Note that hypoxia, either with or without monocrotaline treatment, significantly decoupled the expression coordination of these genes. Citrate cycle: *Acly* (ATP citrate lyase), *Aco2* (aconitase 2), *Fh* (fumarate hydratase), *Idh2* (isocitrate dehydrogenase 2 (NADP+)), *Idh3g* (isocitrate dehydrogenase 3 (NAD), gamma), *Ogdh* (oxoglutarate (alpha-ketoglutarate) dehydrogenase), *Pck2* (phosphoenolpyruvate carboxykinase 2), *Pdha1* (pyruvate dehydrogenase (lipoamide) alpha 1). Fructose and mannose metabolism: *Aldoa/c* (aldolase A/C, fructose-bisphosphate), *Fpbp1* (fructose-1,6-bisphosphatase 1), *Hk1* (hexokinase 1), *Khk* (ketohexokinase), *LOC500959* (triosephosphate isomerase), *Pfkfb3* (6-phosphofructo-2-kinase/fructose-2,6-biphosphatase 3), *Pfkm* (6-phosphofructo-2-kinase/fructose-2,6-biphosphatase 3), *Pmm1/2* (phosphomannomutase 1/2), *Tpi* (triosephosphate isomerase 1). Glycolysis/gluconeogenesis pathway: *Acss2* (acyl-CoA synthetase short-chain family member 2), *Adh1/5* (alcohol dehydrogenase 1/5), *Aldh2* (aldehyde dehydrogenase 2 family), *Aldh3/9a1* (aldehyde dehydrogenase 3/9 family, member A1), *Aldoa/c*, *Eno1/2/4* (enolase 1/2/4), *Fbp1*, *G6pc3* (glucose 6 phosphatase, catalytic, 3), *Gapdh* (glyceraldehyde-3-phosphate dehydrogenase), *Gapdhs* (glyceraldehyde-3-phosphate dehydrogenase, spermatogenic), *Gck* (glucokinase), *Gpi* (glucose-6-phosphate isomerase), *Hk1*, *Ldha/b* (lactate dehydrogenase A/B), *Minpp1* (multiple inositol-polyphosphate phosphatase 1), *Pck2*, *Pdha1*, *Pfkl/m* (Phosphofructokinase, liver/muscle), *Pgam1* (phosphoglycerate mutase 1), *Pgk1* (phosphoglycerate kinase 1), *Tpi* (triosephosphate isomerase 1). Pyruvate metabolism: *Acss2*, *Acyp2* (acylphosphatase 2), *Adh1/5*, *Aldh2*, *Aldh9a1*, *Fh*, *Ldha/b*, *Pck2* (phosphoenolpyruvate carboxykinase 2), *Pdha1*.

Figure 7 presents the expression intercoordination of 40 genes involved in four KEGG-determined metabolic pathways: citrate cycle [42], fructose and mannose [43], glycolysis/gluconeogenesis [41], and pyruvate [44] pathways.

### 3.8. Regulation of Key Genes That Control Mitochondrial Membrane Potential and May Be Responsible for PAH Channelopathies

**Figure 8:**
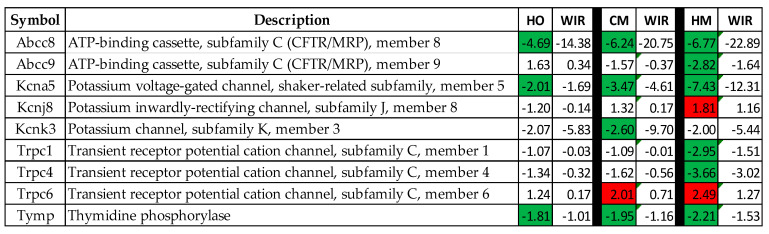
Regulations and the WIR contributions to the lung transcriptomic alteration of nine key genes in HO, CM, and HM groups with respect to CO. Green/red background in the HO, CM, and HM columns indicates that the gene was (*p* < 0.05) significantly regulated according to the “CUT” criterion. Blank background indicates that the regulation was not statistically significant.

Several recent reports (e.g., [45,46,47,48]) discuss the roles played by ionic channels and mitochondrial membrane potential controllers in vascular muscle tone and homeostasis. Figure 8 presents the regulations and the WIR contributions to the lung transcriptomic alteration of nine key genes. Note that *Abcc8* had the largest WIR contribution in all three PAH models.

## 4. Discussion

In this report, we present the effects of pulmonary arterial hypertension on the expression of several important genes and proteins, and on the topology of certain metabolic pathways. The transcriptomic analyses were carried on from the perspective of the Genomic Fabric Paradigm [49] that characterizes the transcriptome by the average expression levels of the composing genes, the control of the transcripts’ abundances, and the expression intercoordination of the genes. In order to verify the independence of the three groups of measures, let us consider the genes A, B, and C with normalized expression levels in four biological replicates: A (96, 98, 102, 104), B (87, 83, 110, 120), C (105, 102, 98, 95). All three genes have the same AVE = 100, but different coefficients of variation: CV_A_ (3.65%), CV_B_ (17.87%), CV_C_ (4.40%), and different coefficients of correlation: COR (A, B) = 0.95 (significant synergism), COR (A, C) = −0.997 (significant antagonism). Another gene D (103, 97, 97, 103), with the same AVE = 100 and similar CV_D_ = 3.46%, is independently expressed with gene A.

Interestingly, the reported Cav1 overexpression in [25] by 39.14x in CM, 13.18x in HO, and 79.06x in HM is qualitatively in line with the overexpression of nerve growth factor (Ngf) in the three models depicted in Figure 1. The results indicate that Cav1 is associated with Ngf. However, an inverse relationship between the protein levels of Cav1 and Ngf was found in the heart of a rat model of induced chronic ischemic heart failure compared to the corresponding control [50]. An increase of Ngf expression associated with Cav1 decrease was reported in the urinary bladder of rats following acute urinary retention after parturition [51]. Our results indicate that expressions of Cav1 and Ngf might be controlled by potentially different upstream factors.

We have added the analyses on Cav1 because this protein was recently reported to play important roles in the glucose metabolic network [52], lipid metabolism [53], mitochondrial metabolism [54], and in cellular metabolic reprogramming [55]. There is also experimental evidence regarding the role of Ngf in cellular energy homeostasis [56].

Although none of the hypoxia exposure and monocrotaline treatments applied alone had practically any effect on the abundance of nuclear factor erythroid-derived 2-like 2 (Nfe2l2), together these two PAH inducers triggered a significant increase of Nfe2l2. We interpret the substantial increase of Nfe2l2 in the HM rats as a desperate activation of a molecular mechanism aiming to maintain the redox balance [57,58,59].

Although we found that hypoxia is the main trigger of the increase in expression of glucose transporter solute carrier family 2 member 1 (Slc2a1), with monocrotaline administration also having a positive effect, other groups [60] gave more credit to monocrotaline. A significant upregulation of the *SLC2A1* gene (also known as *Glut1*) was also reported recently in a group of six patients with end-stage PAH [61].

While checking the 50 immune inflammatory response genes to illustrate the independence of the three types of transcriptomic characteristics, we found that the expression intercoordination of these genes was significantly decreased by PAH in all three models. This result indicates (for the first time to our knowledge) a substantial desynchronization of the expressions of these genes, making the immune inflammatory response more chaotic, which requires the use of anti-inflammatory therapeutics [62]. For now, we have no explanation of why hypoxia alone is the most efficient decoupling factor (group HO, Figure 5), albeit monocrotaline administration also has a significant effect in CM, while reducing the consequences of hypoxia in HM group. Nonetheless, this analysis indicated the powerful impact of PAH on gene networking. We have analyzed the immune response genes owing to the role of the pyruvate metabolism in activating the mitochondrial quality control and inflammation [63].

The traditional percentage of significantly regulated genes has the major handicaps of being based on arbitrarily introduced cutoffs for expression ratio and *p*-value, and considering all regulated genes as equal +1 or −1 contributors. Therefore, we instead used the weighted individual (gene) regulation (WIR) and the transcriptomic distance (TD). WIR accounts for the total change of the expression level, while TD considers the changes in all three types of the gene expression characteristics: the average expression level, the relative expression variability among biological replicates, and the expression coordination with all other genes. As such, WIR and, much more, TD are far better measures of the contributions of individual genes to the transcriptome alteration. For instance, the most significantly upregulated gene in HM, chemokine (C-C motif) ligand 21 (*Ccl21*), has the expression ratio x = 247.07, WIR = 199.89, and TD = 22.88. Each quantifier (excepting the uniform contribution) can be used to establish the regulation hierarchy of the genes, but the resulting hierarchies are not the same. Thus, the most affected three genes in HO, as fold-change, are: *Ccl6* (chemokine (C-C motif) ligand 6, x = 7.53), *Ifngr2* (chemokine (C-C motif) ligand 6, x = −6.97), and *Il34* (interleukin 34, x = 5.71). As WIR, the top three affected genes in HO are: *Il2rg* (interleukin 2 receptor, gamma, WIR = −391), *Tnfsf12* (tumor necrosis factor ligand superfamily member 12, WIR = −222), and *Ifngr2* (WIR = −82.5). As TD, the hierarchy in HO is: *Cx3cr1* (chemokine (C-X3-C motif) receptor 1, TD = 15.90), *Ccrl2* (chemokine (C-C motif) receptor-like 2, TD = 14.60), and *Il2rg* (TD = 9.29). As expected, the hierarchy of the individual gene contributions to the overall transcriptomic alteration also depends on how PAH was installed. With respect to the TD measure, the most affected genes in CM were: *Cx3cr1* (TD = 17.70), *Il2rg* (TD = 10.80), and *Ccl21* (chemokine (C-C motif) ligand 21, TD = 8.17). In HM they were: *Ccl21* (TD = 22.88), *Ifitm2* (interferon induced transmembrane protein 2, TD = 13.00), and *Il2rg* (TD = 9.64).

*Ccl21*, found by us to be upregulated by 3.26x in HO, 79.95x in CM, and 247.09 in HM, has a disputed value as a potential PAH biomarker in systemic sclerosis [64,65]. Nonetheless, our results indicate that the contribution of *Ccl21* to the PAH phenotype depends on the disease etiology, with hypoxic PAH being the least dependent on it according to the transcriptomic distance criterion (TD_HO_ = 4.45, TD_CM_ = 8.05, TD_HM_ = 22.914).

From a transcriptomic point of view, glycolysis/gluconeogenesis was the most affected functional pathway. As presented in Figure 7d, the average TD was 62.84 for HO rats, 55.13 for CMs, and 91.10 for HMs, substantially larger than the average TD of the 50 immune inflammatory genes from Figure 5d: 2.42 (HO), 2.98 (CM), and 2.76 (HM). Glycolysis/gluconeogenesis also tops the list of altered pathways by the percentages of significantly regulated genes in all three PAH models: 42.37% (HO), 55.93% (CM), and 83.05% (HM). Our results confirm that the aberrant glycolysis is a major pathogenic mechanism in the development of PAH [66]. The citrate cycle was also one of the most affected, with 23.33% of the genes significantly regulated in HO, 63.33% in CM, and 70% in HM.

As presented in Figure 8, we found that PAH had a strong impact on the networking of the selected genes. Thus, out of 780 gene pairs, 316 (i.e., 40.5%) were (*p* < 0.05) significantly synergistically expressed in CO. The number of synergistic pairs was reduced to 79 (10.1%) in HO, 260 (33.3%) in CM, and 138 (17.7%) in HM, indicating massive decoupling of the genes in the studied metabolic pathways. There are some remarkable results at the level of individual genes. For instance, the most coupled genes in CO group are *Adh5*, *Pck2*, and *Pmm2*, all with 28 (i.e., 71.8%) synergistically expressed partners within the selection, while *Acyp2*, *Aldh2*, *Eno4*, *Fbp1*, *Fh*, *Gapdhs*, *Pgk1*, and *Pmm1* have no synergistic partners. Our findings confirm the major roles played by *Adh5* (also known as Gsnor [67], *Pck2* [68], and *Pmm2* [69]) for the lung and other organs’ pathophysiology.

In HO, the most coupled genes are *Aldh3a1* and *Gapdh*, with only 10 (25.5%) synergistic partners, while the uncoupled genes (no partners) are *Acss2*, *Acyp2*, *Eno4*, *Gapdhs*, and *Pfkl. Aldh3a1* is a putative biomarker of lung cancer [70], while *Gapdh* was reported as critical for stem cell therapy of pulmonary hypertensive females [71].

The results regarding phosphomannomutases 1 and 2 are also very interesting. The uncoupled *Pmm1* in CO has 9 (23.1%) synergistic partners in HO, 23 (59.0%) in CM, and 10 (25.5%) in HM, while the high interconnection (71.8%) of *Pmm2* in CO is reduced to 7.7% in HO, 17.9% in CM, and 25.5% in HM. These results suggest that PAH replaced *Pmm2* with *Pmm1* in the center of the fructose and mannose metabolism. While *Pmm1* was significantly upregulated in all three PAH models (by 11.87x in HO, 17.71x in CM, and 26.04x in HM), *Pmm2* was significantly downregulated in all three (by −4.65x in HO, −3.50x in CM, and −3.14x in HM). Another surprising finding was the switch from 46.2% synergistic partners and 0% antagonistic ones for *Aldh3a1* in CO, to 0% synergism and 51.3% antagonism in CM, although in the other two PAH models, it was only synergistically connected; in all three models it was significantly downregulated.

In a previous paper [30], we discussed the roles of various ion transporters in the wall myocardia of each heart chamber of adult male mice. The present study on rat lung transcriptomics revealed that the expressions of several ion transporters are regulated in PAH, with monocrotaline treatment being the most disturbing factor.

## 5. Conclusions and Future Directions

Our data indicate a significant association between metabolism dysregulation and pulmonary arterial hypertension. However, this study needs to be continued with direct wet experiments exploring the alterations of the ATP content, as well as of the mitochondrial activity and glycolysis. It will also be very interesting to study the interplay of the metabolic pathway with membrane transporters, and explore common therapeutic avenues for PAH and heart channelopathies.

COVID-19-induced pulmonary vasculopathy looks similar to that of pulmonary arterial hypertension, suggesting that PAH may also be a long-term sequela of SARS-CoV-2 [72,73]. Since both PAH and SARS-CoV-2 cause significant pulmonary injury, in which metabolism dysregulation plays a pivotal role, it will be interesting to study whether the two diseases have similar effects on other organs [74], and whether they share similar therapeutic solutions.

## Data Availability

This study used the gene expression results deposited and publicly available at https://www.ncbi.nlm.nih.gov/geo/query/acc.cgi?acc=GSE72707 (accessed on 1 April 2023).
